# Use of F-18 FDG PET/CT Through Delayed Diuretic Imaging for Preoperative Evaluation of Upper Urinary Tract-Occupying Lesions

**DOI:** 10.3389/fonc.2021.699801

**Published:** 2021-08-30

**Authors:** Jun Wang, Liang Zhang, Jian Guo Wu, Ruohua Chen, Jia lin Shen

**Affiliations:** ^1^Department of Interventional Oncology, Ren Ji Hospital, School of Medicine, Shanghai Jiao Tong University, Shanghai, China; ^2^Department of Nuclear Medicine, Second Affiliated Hospital, Nanchang University, Nanchang, China; ^3^Department of Nuclear Medicine, Ren Ji Hospital, School of Medicine, Shanghai Jiao Tong University, Shanghai, China

**Keywords:** PET/CT, upper urinary tract-occupying lesions, SUVmax, PET, FDG

## Abstract

**Purpose:**

To evaluate the value of F-18 FDG PET/CT in the differentiation of malignant and benign upper urinary tract-occupying lesions.

**Patients and Methods:**

64 patients with upper urinary tract-occupying lesions underwent F-18 FDG PET/CT at RenJi Hospital from January 2015 to February 2019 in this retrospective study. Of the 64 patients, 50 patients received nephroureterectomy or partial ureterectomy; 14 patients received ureteroscopy and biopsy. The comparisons of PET/CT parameters and clinical characteristics between malignant and benign upper urinary tract-occupying lesions were investigated.

**Results:**

Of the 64 patients, 49 were found to have malignant tumors. Receiver operating characteristic analysis determined the lesion SUVmax value of 6.75 as the threshold for predicting malignant tumors. There were significant associations between malignant and benign upper urinary tract-occupying lesions and SUVmax of lesion (P<0.001), lesion size (P<0.001), and patient age (P=0.011). Multivariate analysis showed that SUVmax of lesion (P=0.042) and patient age (P=0.009) as independent predictors for differentiation of malignant from benign upper urinary tract-occupying lesions. There was a significant difference in tumor size between the positive (SUVmax >6.75) and negative (SUVmax ≤6.75) PET groups in 38 of the 49 patients with malignant tumors.

**Conclusion:**

The SUVmax of lesion and patient age is associated with the nature of upper urinary tract-occupying lesions. F-18 FDG PET/CT may be useful to distinguish between malignant and benign upper urinary tract-occupying lesions and determine a suitable therapeutic strategy.

## Introduction

Lesions occupying the upper urinary tract are a commonly encountered problem in the urinary system and can be caused by several diseases. Identifying the cause of upper urinary tract-occupying lesions is often critical for optimal management and prognostication. Urine cytology is often used to diagnose upper urinary tract-occupying lesions (1, 2). However, its sensitivity for detection of upper urinary tract occupying-lesions is not very high (3–5). Owing to miniaturization and increased scope flexibility, ureteroscopy demonstrates more accurate diagnosis (6–8). However, the accuracy of ureteroscopic biopsy is still inherently limited (8, 9). Further cause for concern is the invasive nature of the procedure. Thus, alternative noninvasive strategies that can distinguish malignant from benign upper urinary tract-occupying lesions are needed. Though computed tomography urography (CTU) has been widely used for diagnosis of upper urinary tract carcinoma, benign diseases could mimic upper urinary tract carcinoma.

F-18 FDG PET was often used to differentiate between benign lesions and malignant tumors (10, 11). However, use of F-18 FDG PET/CT in urinary system has developed slowly due to the uptake of F-18 FDG in urine. Though previous studies showed that delayed diuretic PET/CT could be used in the diagnosis of bladder cancer (12–14), only few studies investigated the value of F-18 FDG PET/CT in upper urinary tract-occupying lesions. Though the diagnostic sensitivity of F-18 FDG PET/CT for detecting metastasis was superior to CT in upper urinary tract carcinoma, it was not feasible to detect the primary tumor because of the radiotracer in the urine (15). The aim of our study was to investigate the value of F-18 FDG PET/CT through delayed diuretic imaging in determining the cause of upper urinary tract-occupying lesions.

## Materials and Methods

### Patient Demographics

In Renji Hospital delayed diuretic F-18 FDG PET/CT were routinely performed in patients with upper urinary tract-occupying lesions. 64 patients with upper urinary tract-occupying lesions who underwent F-18 FDG PET/CT were included at RenJi Hospital from January 2015 to February 2019 in this retrospective study. Before F-18 FDG PET/CT, 53 patients underwent contrast-enhanced CT, 7 patients underwent magnetic resonance, 2 patients underwent ultrasound and 2 patients underwent ureteroscopy. Despite the presence of upper urinary tract-occupying lesions on imaging (CT, magnetic resonance, and ultrasound) and ureteroscopy, the nature of the lesion were unclear. F-18 FDG PET/CT was undertaken to clarify the nature of the lesion and total-body condition. The inclusion criteria were: a) the consecutive patients who presented with an upper tract lesion underwent F-18 FDG PET/CT between January 2015 and February 2019; b) the lesions were subsequently histologically diagnosed; c) patients information including gender, age, primary site laterality, lesion site and pathological information were available. Of the 64 patients, 50 patients received nephroureterectomy or partial ureterectomy; 14 patients received ureteroscopy and biopsy. For the 49 had malignant lesions, radical resection was used for patients without metastatic disease and palliative nephroureterectomy was used for patients with metastatic disease. For the 15 patients had benign lesions, 7 patients received biopsy and 8 patients received surgical operation. All lesions were confirmed by pathological diagnosis. The tumor originated in the renal pelvis and the ureter in 31 (48.4%) and 33 (51.6%) patients, respectively. Patient characteristics are listed in **Table 1**. The institutional review board of RenJi Hospital approved this study. The informed consent was waived because it was a retrospective study.

**Table 1:** Patient characteristics.

Characteristics	No. of Patients
**Sex**	
Male	34 (53.1%)
Female	30 (46.9%)
**Age (y)**	
Mean ± SD	64.2 ± 12.2
Range	26-88
**Primary site laterality**	
Right	33 (51.6%)
Left	31 (48.4%)
**Lesion size**	
Mean ± SD	24.0 ± 17.2
Range	4-84
**SUVmax**	
Mean ± SD	14.5 ± 11.6
Range	1.4-46.8
**Lesions**	
Malignant	
Pelvis	27 (42.2%)
Ureter	22 (34.4%)
Benign	
Pelvis	4 (6.3%)
Ureter	11 (17.2%)
Positive lesions	
Malignant	49 (76.6%)
Benign	15 (23.4%)

### F-18 FDG PET/CT Imaging

Blood glucose levels should be less than <140 mg/dL. After a minimum fasting and resting period of 6 h and 1 h, respectively, F-18 FDG was injected for every patient at the dose of 3.7 MBq/kg. F-18 FDG PET/CT scanning was performed using a whole-body scanner (Biograph mCT; Siemens) (early PET/CT imaging). Delayed PET/CT imaging was performed after 120 min of early PET/CT imaging. Patients received intravenous furosemide (20 mg) and oral intake of at least 500 mL water. Delayed imaging covered a range of 1–2 bed positions centered at the location of the lesion. The response of delayed PET/CT imaging was uniform for all patients.

The PET/CT images were evaluated by two experienced nuclear medicine physicians (Yiping Shi and Ruohua Chen). They evaluated the images independently and were blinded to patients’ information. For patients with metastatic disease, the SUVmax of urinary tract lesions were measured. When discrepancies occurred, they reached a consensus. There was perfect agreement between them (k coefficient = 0.96).

### Statistical Analysis

The data are shown as mean ± SD. Statistically significant differences between groups were compared using the chi-square test, Fisher’s exact test, or Mann–Whitney *U* test where applicable. *P* < 0.05 was considered statistically significant. Statistical analyses were performed using SPSS, version 13.0.

## Results

### Patient Characteristics

**Figure 1:**
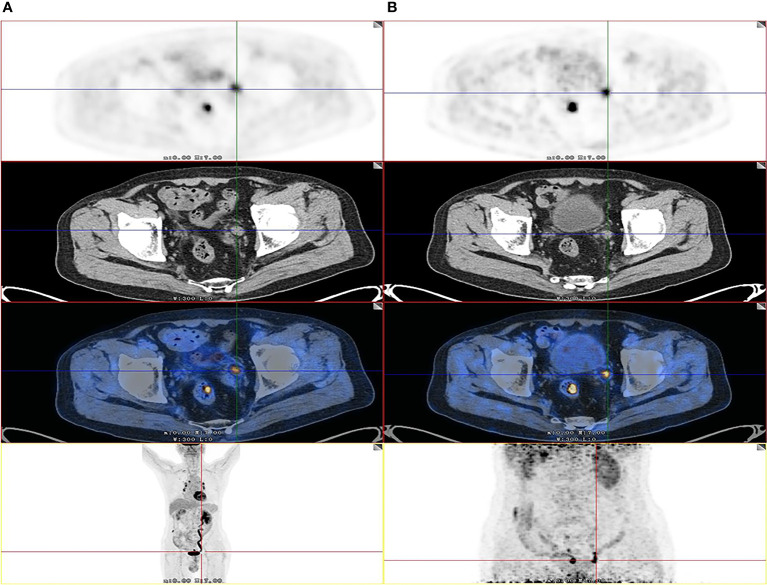
Images of a 82-year-old patient with urothelial carcinoma. On the F-18 FDG PET/CT images, CT show that the thickening was located in left lower ureter. However, the FDG uptake of lesion was unable to detect due to urine interference in the early image **(A)**. On the delayed phase, the ureter is distended and lesion can be easily visualized by axial CT and PET (SUVmax, 12.2) **(B)** as the SUVmax of urine was decreased. The patient received radical ureterectomy, and high-grade papillary urothelial carcinoma was confirmed by histopathology.

Among the 64 patients, 49 had malignant lesions: these included 23 ureteral urothelial carcinoma and 26 pyelurothelial carcinoma. 15 patients had benign lesions, which were confirmed as inflammatory hyperplasia. Of the 49 patients with malignant tumor, 4 had low grade, and 45 had high grade. Of the 49 patients with malignant tumor, 16 had lymph node metastasis, 4 had pulmonary metastasis and 2 had bone metastasis. **Table 1** shows the patient characteristics. The average SUVmax of urine of the early image was 32.9 ± 30.0. Due to the radiotracer in the urine, it was not feasible to measure the SUVmax of the lesion on the early images. On the delayed F-18 FDG PET/CT, the ureter is distended and lesion can be easily measured. The SUVmax of urine of the delay image was 3.5 ± 1.6. A patient with ureteral carcinoma who underwent F-18 FDG PET/CT through delayed diuretic imaging was shown in **Figure 1**. The average SUVmax of malignant and benign upper urinary tract lesions were 17.5 ± 11.6 and 4.6 ± 2.8, respectively. There was no difference in SUVmax between low grade ureteral carcinoma and high grade ureteral carcinoma (16.6 ± 5.8 *vs*. 19.0 ± 1.7, P= 0.809).

The rates of positive F-18 FDG uptake were significantly higher in malignant than in benign upper urinary tract lesions [93.9% (46/49) *vs*. 53.3% (8/15), respectively; *P* = 0.001], when compared with normal liver tissues.

### Differences Between Malignant and Benign Upper Urinary Tract Lesions

The relationship between patients’ characteristics and malignant or benign upper urinary tract lesions were shown in **Table 2**. No significant differences were observed in lesion site, primary site laterality. However, there were significant differences in SUVmax, age, and lesion size. Malignant lesions showed significantly higher SUVmax than benign lesions (17.5 ± 11.6 *vs*. 4.6 ± 2.8; *P* < 0.001). No significant difference were found in the SUVmax between ureteral urothelial carcinoma and pyelurothelial carcinoma (15.8 ± 12.6 *vs*. 19.1 ± 10.6; *P* = 0.232).

**Table 2:** Patient characteristics according to malignant or benign upper urinary tract lesions.

Characteristics		Total (n=64)	Malignant tumors (n=49)	Benign lesions (n=15)	P
**Age**			67.0 ± 9.5	54.9 ± 15.7	0.011
**Sex**					
Male		34	28	6	0.244
Female		30	21	9	
**Primary site laterality**					
Right		33	25	8	0.875
Left		31	24	7	
**Lesion Site**					0.054
Pelvis		31	27	4	
Ureter		33	22	11	
**Lesion Size**			27.6 ± 17.3	12.1± 10.5	< 0.001
**SUVmax**			17.5 ± 11.6	4.6 ± 2.8	< 0.001

### SUVmax Cut-Off Value

**Figure 2:**
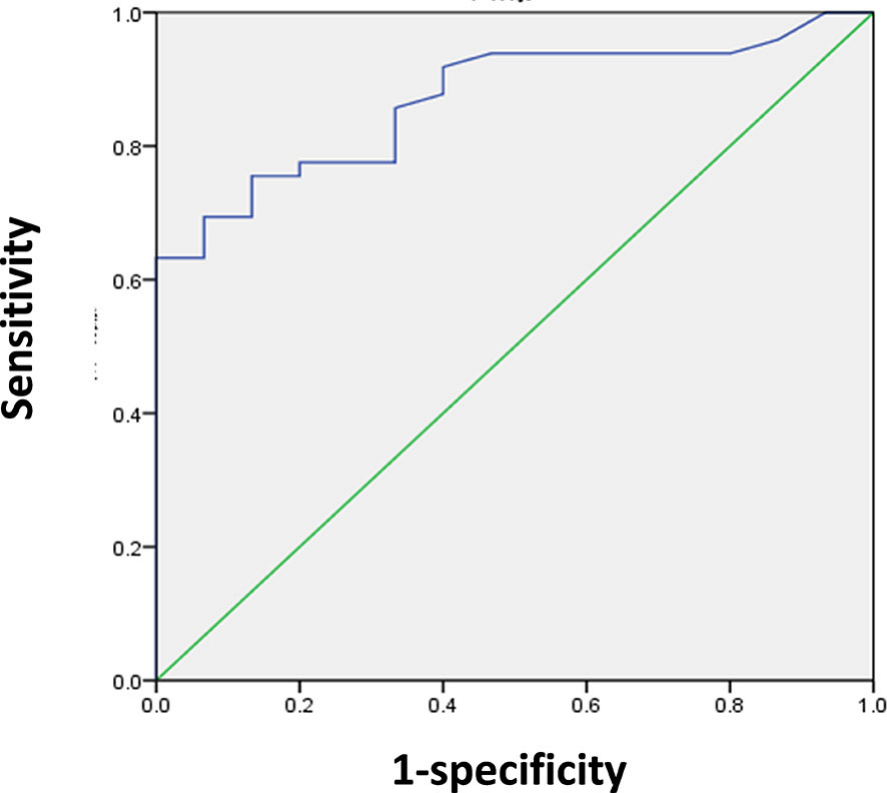
ROC analysis for the differentiation of malignant from benign upper urinary tract occupying in 64 patients. The area under curve was 0.870 (95%CI 0.783–0.957, P < 0.001), and 6.75 was the optimal SUVmax for predicting malignant lesions. When SUVmax of lesion was 6.75, sensitivity and specificity for the prediction of malignant lesions were 75.5% and 86.7%, respectively.

We determined the SUVmax threshold for optimal differentiation between malignant and benign lesions. Receiver-operating-characteristic curve (ROC) analysis revealed that the highest accuracy (78.1%) was obtained with the SUVmax of 6.75 and the area under curve was 0.870 ± 0.045 (**Figure 2**). When SUVmax was 6.75, the sensitivity, specificity, positive predictive value (PPV), and negative predictive value (NPV) for the prediction of malignant tumors were 75.5% (37/49), 86.7% (13/15), 94.8% (37/39), and 52.0% (13/25), respectively. The ROC analysis also identified the optimal cut-off values as 13.5 mm for lesion size and 58 years for age.

### Prediction of Malignant Tumors

Multivariate analysis indicated that SUVmax of the lesion (*P* = 0.042, 95% CI: 1.1–54.7) and age (*P* = 0.009, 95% CI: 1.8–48.1) were independent predictors for malignant lesions in patients with ureteral-occupying lesions, whereas lesion size was not (*P*=0.105, 95% CI: 0.7–28.9) (**Table 3**).

**Table 3:** Multivariate analysis of malignant tumors in patients with ureteral occupying.

Factors	Odds Ratio	OR(95% CI)	P
SUVmax	7.7	1.1 - 54.7	0.042
age	9.2	1.8 - 48.1	0.009
lesion size	4.6	0.7 - 28.9	0.105

Next, we classified the patients into three groups according to their potential for having malignant lesions: high-potential group (SUVmax > 6.75 and age > 58 years), moderate-potential group (SUVmax > 6.75 and age ≤ 58 years, or SUVmax ≤ 6.75 and age > 58 years), and low-potential group (SUVmax ≤ 6.75 and age ≤ 58 years). The probability of having malignant tumors in these groups was 97.0% (32/33), 75.0% (15/20), and 18.2% (2/11), respectively (*P* < 0.001; **Table 4**).

**Table 4:** Rate of malignant tumors in patients with ureteral occupying with high, moderate, and low potential, as indicated by SUVmax and lesion size (n=64).

Potential	Total	Malignant tumors (%)		P
		Positive	Negative	
High	33	97.0	3.0	<0.001
Moderately	20	75.0	25.0	
Low	11	18.2	81.8	
Total	64	76.7	23.4	

### The Association Between Patient Characteristics and PET/CT In Patients Treated With Ureterectomy or Nephrectomy

Of the 49 patients with malignant tumors, 38 were treated with nephroureterectomy or partial ureterectomy. We investigated the relationship between patient characteristics and PET/CT parameters in the 38 patients (**Table 5**). The patients were classified into two groups based on the SUVmax of malignant lesions: positive PET group (SUVmax > 6.75) and negative PET group (SUVmax ≤ 6.75). There were no significant difference between age, sex, tumor laterality, tumor site, T stage, N stage and positive/negative group. However, the positive PET group had larger tumor size than the negative PET group (30.8 ± 17.2 *vs*. 11.3 ± 4.7; *P* < 0.001).

**Table 5:** Patient characteristics according to positive and negative PET group (n=38).

Characteristics	Total (n=38)	Positive	Negative	P
**Age**		68.4 ± 10.3	65.5 ± 9.1	0.423
**Sex**				
Male	23	16	7	0.288
Female	15	11	4	
**Tumor laterality**				
Right	20	14	6	0.721
Left	18	13	5	
**Tumor Site**				0.061
Pelvis	24	20	4	
Ureter	14	7	7	
**Tumor Size (mm)**		30.8 ± 17.2	11.3 ± 4.7	<0.001
**T stage**				
<T3	17	10	7	0.167
≥T3	21	17	4	
**N stage**				0.195
**0**	30	23	7	
**1**	8	4	4	

## Discussion

F-18 FDG PET/CT is commonly used for diagnosis of many malignant tumors (16–18). However, FDG is not widely used in urology due to its urinary elimination (13). In this study, the value of F-18 FDG PET/CT for diagnosing upper urinary tract-occupying lesions was investigated. This study was the first to evaluate whether F-18 FDG PET/CT could be used for differentiating benign from malignant upper urinary tract-occupying lesions.

Upper urinary tract-occupying lesions are known to occur due to various diseases such as urothelial carcinomas or inflammatory hyperplasia (7). Although ureteroscopy and biopsy are commonly used for the diagnosis of benign and malignant lesions, its accuracy for upper urinary tract occupying-lesions is limited; furthermore, ureteroscopy is an invasive procedure that carries the risk of infection (19, 20). In this study, we found that F-18 FDG PET/CT could be used to differentiate malignant from benign upper urinary tract-occupying lesions. The ROC analysis indicated that SUVmax can be used for predicting whether the lesion is benign or malignant. Multivariate analysis revealed that both the SUVmax of the lesion and age were independent predictors of the nature of the lesion. This is the first study to indicate that the SUVmax of upper urinary tract-occupying lesions could differentiate between benign and malignant lesions. When SUVmax was 6.75, the sensitivity and specificity for predicting malignant lesions were 75.5% (37/49) and 86.7% (13/15), respectively. In addition, we also found that patient age were significant predictors for predicting malignant lesions, which was consistent with previous studies.

We categorized patients with upper urinary tract-occupying lesions into high-potential, moderate-potential, and low-potential groups based on their potential for having malignant lesions, as indicated by SUVmax and age. The chance of having malignant lesions detected in 97.0% of the patients in the high-potential group, but only in 18.2% of the patients in the low-potential group. These data suggest that the possibility of malignancy is very high in patients classified to the high-potential group; these findings merit further attention.

In this study, we also investigated the relationships between SUVmax of tumors and clinicopathologic features in 38 patients with malignant lesions. Our findings suggest that upper urinary tract urothelial carcinomas with high FDG accumulation are likely larger tumors. Therefore, when using SUVmax to distinguish malignant from benign lesions, the tumor size should also be considered. However, tumor size was not the independent predictor for malignant tumors in patients with ureteral occupying.

Our study had several limitations because it was a retrospective study and had small samples. In our study 14 patients were confirmed only by ureteroscopy and biopsy, and they have limitations in upper tract disease. Previous studies showed that micropapillary and sarcomatoid variants were associated with poorer oncological outcomes, and molecular markers such as human epidermal growth factor receptor−2 (HER2) is considered to be a potential prognostic and therapeutic marker in patients with upper tract urothelial carcinoma (21, 22). In our study, no patients with micropapillary and sarcomatoid variants were found. They might be because our sample was small. In addition, molecular markers were not detected in our study, so the relationship between molecular markers and prognosis were not analyzed. Further prospective studies with large samples and molecular markers are needed to confirm our results.

## Conclusions

Our study was the first to investigate the diagnostic value of F-18 FDG PET/CT in the upper urinary tract system and demonstrated that F-18 FDG PET/CT through delayed diuretic imaging could be used for differentiating malignant from benign upper urinary tract-occupying lesions. Our study may advance the development of noninvasive strategies to predict the cause of upper urinary tract-occupying lesions.

## Data Availability Statement

The raw data supporting the conclusions of this article will be made available by the authors, without undue reservation.

## Ethics Statement

The studies involving human participants were reviewed and approved by Renji Hospital. Written informed consent for participation was not required for this study in accordance with the national legislation and the institutional requirements.

## Author Contributions

JS and RC designed and wrote the experiments. JW, LZ, and JG collected and analyzed the data. All authors contributed to the article and approved the submitted version.

## Funding

The study was supported by National Natural Science Foundation of China (nos. 81701724) and JiangXi Provincial Department of Science and Technology (Grant Number: 20161BBG70202 & 20071BBG70050), Jiangxi Provincial Health Commission (Grant Number: 20161071 & 20171090).

## Conflict of Interest

The authors declare that the research was conducted in the absence of any commercial or financial relationships that could be construed as a potential conflict of interest.

## Publisher’s Note

All claims expressed in this article are solely those of the authors and do not necessarily represent those of their affiliated organizations, or those of the publisher, the editors and the reviewers. Any product that may be evaluated in this article, or claim that may be made by its manufacturer, is not guaranteed or endorsed by the publisher.
